# The protein binding substance Ibuprofen does not affect the T1 time or partition coefficient in contrast-enhanced cardiovascular magnetic resonance

**DOI:** 10.1186/1532-429X-14-71

**Published:** 2012-10-15

**Authors:** Nadine Kawel, Francesco Santini, Tanja Haas, Johannes M Froehlich, Jens Bremerich

**Affiliations:** 1 Department of Radiology, University Hospital Basel, Petersgraben 4, Basel 4031, Switzerland; 2 Radiological Physics, University Hospital Basel, Petersgraben 4, Basel 4031, Switzerland; 3 Scientific Affairs, Guerbet Switzerland, Winterthurerstrasse 92, Zurich 8006, Switzerland

**Keywords:** Gadobenate dimeglumine, Ibuprofen, Interaction, CMR, Diffuse myocardial fibrosis

## Abstract

**Background:**

Contrast enhanced cardiovascular magnetic resonance (CMR) with T1 mapping enables quantification of diffuse myocardial fibrosis. Various factors, however, can interfere with T1 measurements. The purpose of the current study was to assess the effect of co-medication with a typical protein binding drug (Ibuprofen) on T1 values in vitro and in vivo.

**Methods:**

50 vials were prepared with different concentrations of gadobenate dimeglumine, Ibuprofen and human serum albumin in physiologic NaCl solution and imaged at 1.5T with a spin echo sequence at multiple TRs to measure T1 values and calculate relaxivities. 10 volunteers (5 men; 31±6.3 years) were imaged at 1.5T. T1 values for myocardium and blood pool were determined for various time points after administration of 0.15mmol/kg gadobenate dimeglumine using a modified look-locker inversion-recovery sequence before and after administration of Ibuprofen over 24 hours. The partition coefficient was calculated as ΔR1_myocardium_/ΔR1_blood_, where R1=1/T1.

**Results:**

In vitro no significant correlation was found between relaxivity and Ibuprofen concentration, neither in absence (r=−0.15, p=0.40) nor in presence of albumin (r=−0.32, p=0.30). In vivo there was no significant difference in post contrast T1 times of myocardium and blood, respectively and also in the partition coefficient between exam 1 and 2 (p>0.05). There was good agreement of the T1 times of myocardium and blood and the partition coefficient, respectively between exam 1 and 2.

**Conclusions:**

Contrast enhanced T1 mapping is unaffected by co-medication with the protein binding substance Ibuprofen and has an excellent reproducibility.

## Background

It has been demonstrated that T1 time as measured by T1 mapping by means of cardiovascular magnetic resonance (CMR) correlates with histologically proven fibrosis.[1] Post-contrast T1 values have been shown to be altered in various cardiac diseases such as systemic lupus erythematosus [2], cardiac amyloidosis [3], chronic aortic regurgitation [4], adult congenital heart disease [5], and diabetic cardiomyopathy [6,7]. Since the concentration of the gadolinium based contrast agent is directly related to the difference between pre-contrast and post-contrast reciprocal values of T1 (ΔR1) and based on the assumption of a steady state of the concentration of the gadolinium based contrast agent between the extracellular space and the blood pool, the extracellular volume fraction (ECV) which is directly related to the collagen content can be quantified by calculating the ratio of pre- and post-contrast reciprocal values of T1 measured in blood and myocardium corrected for the hematocrit [ECV = ΔR1myocardium/ΔR1blood * (1-hematocrit)]. Calculation of the partition coefficient (Lambda) is identical to the ECV except that it is not corrected for the hematocrit. Messroghli et al. demonstrated a correlation between ECV and the collagen volume fraction quantified by histologic analysis.[8] Wong et al. recently identified the predictive value of expansion of the extracellular matrix as calculated by ECV and all cause mortality as well as a composite end point of death, cardiac transplant and left ventricular assist device. [9] T1 mapping and calculation of the imaging biomarker ECV and the partition coefficient, respectively is a promising technique that might replace invasive myocardial biopsy in diagnosing diffuse myocardial fibrosis and might be a useful tool to monitor therapy. Advantages of CMR T1 mapping as compared to myocardial biopsy are 1) non-invasiveness and therefore the possibility to repeat measurements for treatment monitoring, 2) large measurement volume rather than small biopsy samples and 3) the fact that no ionizing radiation is required.

T1 mapping is technically demanding since technical, physiological, and biochemical factors can interfere with T1 measurements. For example renal function as reflected by glomerular filtration rate is of relevance [10]. Moreover, contrast material dose, relaxivity, biodistribution, clearance, interaction with plasma proteins, and interference with co-medication must be taken into consideration.

It has been demonstrated that both the T1 relaxation time and the partition coefficient lambda (calculated by the change in relaxation rate of blood and myocardium) vary with relaxivity and distribution properties of the contrast material [11]. Gadobenate dimeglumine (Gd-BOPTA, Multihance®) has some weak protein binding capacities and higher molar relaxivity in plasma/blood as compared to other extracellular gadolinium based contrast agents leading to a shorter T1 time [12-14]. Indirectly, rise of molar relaxivity is linked to protein interaction of the gadolinium based contrast agent influencing also its distribution with reduction of extravasation or prolongation of elimination half-time as shown with gadofosveset trisodium [15]. Other gadolinium based contrast agents also have protein binding capacity but to a lesser extent [16].

Hypothetically co-administration of another drug with a high protein binding capacity might compete with Gd-BOPTA and reduce its bound fraction, therefore altering T1 time. It is well known in pharmacology that albumin binding drugs potentially can interfere with each other due to this interaction with the binding sites on the albumin. This can also be explained by the fact, that the bound fraction (often > 99%) usually is not active while the non-bound fraction (1%) is active and can extravasate. Both effects might as well play a role when studying the relaxivity effects with Gd-BOPTA and the T1 mapping. From our perspective these potential interactions must be studied more in detail and are of high clinical interest, both from a mechanistic understanding but also clinically speaking.

Therefore the aim of this study was to evaluate the interference of a typical protein binding drug (Ibuprofen) with Gd-BOPTA with respect to T1 times in-vitro and in-vivo.

Ibuprofen was chosen since it is a common drug in widespread use available without prescription and known to have a high protein binding capacity. Moreover, the dosage in mg and moles compared to other non-steroidal anti-inflammatory drugs is quite high.

## Methods

### In vitro sample preparation

A total of 50 vials were prepared by diluting Gd-BOPTA (Gadobenate dimeglumine, Multihance, Bracco Imaging, Milan, Italy), Ibuprofen and human albumin in physiologic NaCl solution. The vials contained all combinations of the following dilutions: Gd-BOPTA corresponding to Gadolinium concentrations of 0, 2, 4, 8 and 16mmol/l, Ibuprofen in concentrations of 0, 100mg/l (=0.48mmol/l), 200mg/l (=0.97mmol/l), 400mg/l (=1.94mmol/l) and 1000mg/l (=4.85mmol/l), Albumin in concentrations of 0 and 4g/dl (according to the physiologic concentration of human serum albumin of adults).

### Study subjects

10 volunteers (5 men; mean age ± SD, 31 ± 6.3 years) were included in the in vivo part of the study. All volunteers were healthy subjects without a known cardiovascular disease or systemic conditions and were not on regular medication. All volunteers had a normal creatinine value indicating a normal renal function and no signs of a dyslipoproteinemia as assessed by serum electrophoresis and immunfixation for Bence-Jones proteins in urine. All study participants signed informed consent in this institution review board approved study.

### Image acquisition – in vitro

Samples were placed in a water container and imaged on a 1.5T clinical magnet (Avanto, Siemens Medical Solutions, Erlangen, Germany) in order to establish T1 values. Samples were scanned with a coronal 2D spin echo sequence at multiple TRs with the following parameters: TE 6.2ms; bandwidth 781Hz/pixel; slice thickness 10 mm; field of view 300 x 206 mm; matrix 256 x 176; TR 20, 25, 30, 40, 50, 75, 100, 150, 200, 250, 300, 400, 500, 1000, 1500, 2000, 4000 ms.

### Image acquisition – in vivo

Volunteers were imaged on a 1.5T magnet (Espree, Siemens Medical Solutions, Erlangen, Germany). For T1 mapping a modified look-locker inversion-recovery (MOLLI) sequence was used to acquire images at mid-ventricular short axis pre contrast and every 5 minutes between 5 and 60 minutes after intravenous bolus administration of 0.15mmol/kg Gd-BOPTA. The MOLLI sequence acquired 11 images at different inversion times using the following scan parameters: TE/TR 1.06ms/2.5ms; flip angle 35°; bandwidth 1002Hz/pixel; slice thickness 8mm; field of view 340 x 255 mm; matrix 192 x 138; TI initial 100ms; TI increment 80ms.[17]All volunteers were scanned twice at intervals of 24 hours. Immediately after the first scan 800mg Ibuprofen (Irfen®-800 retard, Mepha Pharma AG, Switzerland) was administered orally and again after 12 hours and 4 hours prior to the second scan. Ibuprofen was taken at least 30 minutes preprandial or 2 hours postprandial.

### Image analysis – in vitro

T1 values were calculated for each sample by a pixelwise fitting of the signal intensities, directly on the scanner, by means of a custom reconstruction procedure [18]. The images were then transferred to a personal computer and circular ROIs were drawn on the sections of the tubes in order to obtain the average T1 values. For each Gd-BOPTA dilution series, the relaxivity value r1 was calculated as the slope of the linear fitting of the inverse of the T1 times, obtaining in the end 10 relaxivity values for Gd-BOPTA in presence of the different Ibuprofen concentrations, with and without albumin.

### Image analysis – in vivo

In vivo T1 maps were generated using MRmap [19] and transferred to QMass V.7.2 (Medis Medical Imaging Systems, Netherlands). Left ventricular endocardial and epicardial contours were drawn manually while segments were defined automatically. T1 time was determined for each segment (American Heart Association segments 7–12 [20]) separately and also calculated for the entire slice as the mean value of all segments excluding segments with severely impaired image quality. T1 time of blood was also measured. Measurements were obtained in the LV cavity taking care to avoid inclusion of the papillary muscles using a region of interest (ROI) covering an area of 3–4 cm^2^. Heart rate correction as it has been suggested for studies using 3T scanners was not performed since differences for shorter T1 times at normal heart rates are very small.[21] The partition coefficient which reflects the change in relaxation rate of myocardium and blood was approximated by ΔR1_myocardium_/ΔR1_blood_, where R1=1/T1 [22].

### Statistical analysis – in vitro

Relaxivity values in presence or absence of albumin were compared by a paired t-test to establish the effect of protein binding on the relaxivity of Gd-BOPTA. Correlation between the calculated relaxivity values and Ibuprofen concentration was evaluated by Pearson’s correlation coefficient, separately in presence or absence of albumin.

### Statistical analysis – in vivo

Statistical analysis was performed using PASW (SPSS) (version 19) and SAS (version 9.2) statistical software. A p-value <0.05 was considered statistically significant.

The continuous variables, T1 time and lambda, are expressed as mean ± SD and were compared using student’s paired t-test for pre contrast values of T1 since data was normally distributed (Kolmogorov-Smirnov test). A linear mixed-model analysis was performed with the log transformed T1 times for blood and myocardium to compare T1 time of both exams after contrast administration. Linear mixed-model analysis with the partition coefficient was also performed. Both sets of models evaluated group, time and group-by-time interaction by study subjects as random effects. Correlation between T1 times and the partition coefficient, respectively acquired in exam 1 and 2 was assessed by calculating the intraclass correlation coefficient (ICC) using a two-way random model and agreement was assessed by generating Bland Altman plots.

## Results

**Figure 1:**
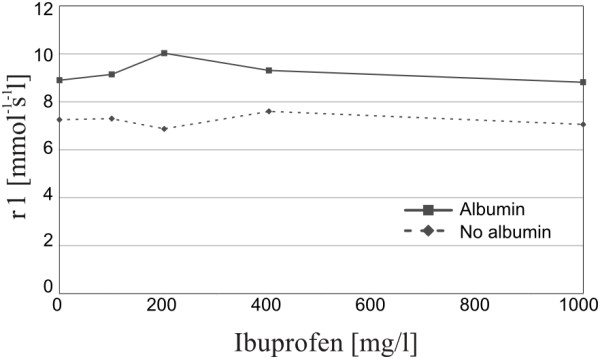
**In vitro experiment**: **Change in T1**-**Relaxivity values with increasing Ibuprofen concentration in presence or absence of albumin.**

T1-Relaxivity values of the in vitro experiment with Gd-BOPTA (Figure 1) were found to be significantly different in presence (9.24 ± 0.48 mmol^-1^s^-1^l) or absence (7.22 ± 0.28 mmol^-1^s^-1^l) of albumin (p=0.001). However, no significant correlation was found between relaxivity and Ibuprofen concentration, neither in absence (R=−0.15, p=0.40) nor in presence of albumin (R=−0.32, p=0.30).

In the in vivo part of the study of 1560 myocardial segments evaluated (6 segments per slice at 13 time points in 10 subjects at 2 exams), 10 segments (0.6%) had to be excluded from analysis related to severely impaired image quality. Mean values ± SD of the in vivo part are shown in Table 1. Pre contrast T1 times for myocardium and blood were not significantly different between exam 1 and 2 (p>0.05). Linear mixed model analysis did not show a group difference between exam 1 and 2 for post contrast T1 values of myocardium, blood and the partition coefficient. Mean T1 times of myocardium and blood and also the mean partition coefficient increased over time (p<0.001) between 5 and 60 minutes after contrast administration. The mean increase of the partition coefficient between 5 and 60 minutes after contrast administration was 19% (exam 1) and 12% (exam 2), respectively.

**Table 1:** **Mean T1 times of myocardium and blood and the partition coefficient** (**lambda**)

	**T1 myocardium** (**ms**)		**T1 blood** (**ms**)		**Lambda**	
**min**	**Exam 1**	**Exam 2**	**Exam 1**	**Exam 2**	**Exam 1**	**Exam 2**
**Pre**	1011 ± 32	1002 ± 48	1526 ± 64	1547 ± 58		
**5**	301 ± 16	308 ± 22	148 ± 16	155 ± 24	0.382 ± 0.03	0.386 ± 0.04
**10**	353 ± 21	367 ± 20	199 ± 25	206 ± 20	0.420 ± 0.04	0.410 ± 0.03
**15**	389 ± 23	367 ± 20	255 ± 24	234 ± 21	0.416 ± 0.03	0.409 ± 0.03
**20**	411 ± 26	403 ± 23	246 ± 29	253 ± 23	0.423 ± 0.03	0.411 ± 0.03
**25**	432 ± 27	425 ± 18	265 ± 30	272 ± 23	0.424 ± 0.03	0.411 ± 0.03
**30**	450 ± 22	445 ± 16	283 ± 31	290 ± 23	0.426 ± 0.03	0.419 ± 0.03
**35**	465 ± 25	460 ± 15	300 ± 33	306 ± 24	0.434 ± 0.03	0.428 ± 0.03
**40**	475 ± 22	479 ± 21	315 ± 33	321 ± 25	0.442 ± 0.04	0.440 ± 0.04
**45**	488 ± 27	500 ± 17	329 ± 35	338 ± 26	0.444 ± 0.03	0.432 ± 0.03
**50**	502 ± 27	509 ± 18	344 ± 37	351 ± 28	0.446 ± 0.04	0.440 ± 0.04
**55**	513 ± 26	525 ± 14	358 ± 38	364 ± 29	0.449 ± 0.04	0.431 ± 0.04
**60**	524 ± 25	537 ± 16	373 ± 38	379 ± 31	0.454 ± 0.04	0.432 ± 0.04

min = time after contrast administration.

**Figure 2:**
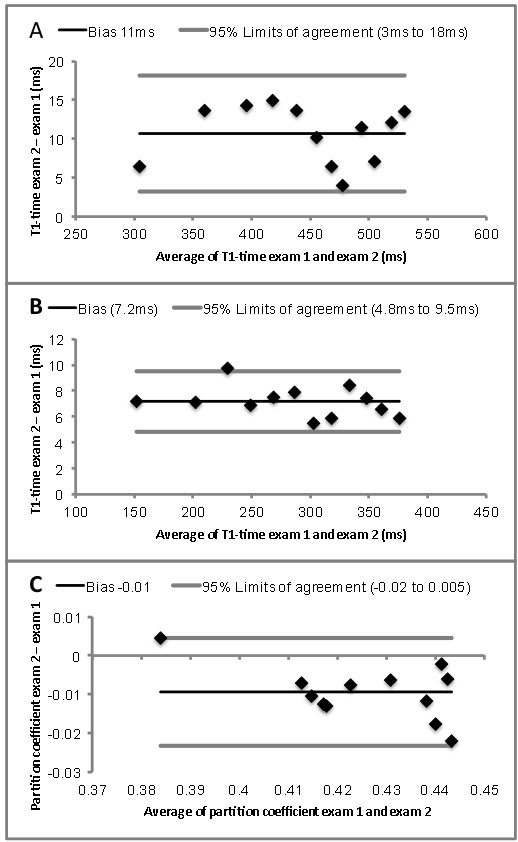
Agreement of T1 times of myocardium (A), blood (B) and the partition coefficient (C), respectively between exam 1 and 2.

The ICC for measurements of T1-time of myocardium and blood, respectively was 1.0 and for the partition coefficient it was 0.82. Bland Altman plots demonstrate a good agreement for the measurements obtained in both exams (Figure 2).

## Discussion

We studied the effect of co-medication with a typical protein binding drug (Ibuprofen) on contrast enhanced T1 mapping. Most important findings can be summarized as follows: 1.) Ibuprofen did not significantly affect T1 times of myocardium and blood when using a gadolinium based contrast agent with protein binding capacity (Gd-BOPTA) 2.) Correlations of T1 times of myocardium and blood and partition coefficient between exam 1 and 2 were excellent.

Injected intravenously the extracellular gadolinium based contrast agent Gd-BOPTA is known to have a transient and weak interaction with plasma proteins, mainly human serum albumin (HSA) [23]. The aromatic tail of Gd-BOPTA enables binding to hydrophobic pockets on the HSA surface [24]. Bound to HSA, the Gd-BOPTA molecule has a lower molecular tumbling rate and a longer rotational MR correlation time resulting in an increased relaxivity [25]. Relaxation rate increases exponentially with increasing HSA concentration in a physiological range [24]. The protein bound fraction of Gd-BOPTA is only about 10% [12].

We hypothesized that the protein bound fraction might be smaller and subsequently relaxivity lower resulting in longer T1 times for myocardium and blood post contrast administration when another drug with protein binding capacity is administered. Results of the in vivo and the in vitro part of the study demonstrated that T1 times were not affected by Ibuprofen. The reason might be a stronger binding to HSA of Gd-BOPTA compared to Ibuprofen. Moreover, it should be emphasized that relaxivity depends on a large variety of dynamic and structural factors which in this experiment could not be further elucidated [26]. In the present case it seems that inner-sphere water exchange which is the most important relaxivity influencing factor is definitely much more affected by ligand-protein interaction than by drug-drug or drug-protein interaction. Our in vitro experiments thus confirm, that there is no clear negative affection of relaxivity even at equimolar concentrations of Ibuprofen and Gd-BOPTA. Further studies are warranted to better understand the dynamics of water-exchange besides hydration of gadolinium complexes in the presence of various protein binding drugs in a proteinous environment. In fact, albumin not only increases the relaxivity of gadolinium complexes in the blood pool, but also emerges as a versatile carrier for a long list of therapeutic and diagnostic agents in pathologies such as diabetes, cancer, rheumatoid arthritis and infectious diseases [27]. Displacement of drugs from their plasma binding increases the unbound drug fraction potentially increasing drug effect or potential toxic effects, so that protein-binding of drugs and drug-drug interactions are of high pharmacological interest. Gadolinium complexes with a high HSA interaction, at least in theory, could be used to visualize such effects, presuming that the drug interferes sufficiently with the Gd-complex – albumin binding sites, which was not the case in the present model.

In the current study we further demonstrated an excellent correlation and good agreement, respectively between the two exams for pre and post contrast T1 values of myocardium and blood, respectively and the partition coefficient that makes it a valuable tool for serial exams and intra-individual comparisons. This result is in agreement with a study by Messroghli et al. who found a high reproducibility of pre and post contrast myocardial T1 times [28].

According to a previous publication, we also detected a statistically significant increase of the partition coefficient over time indicating that no equilibrium establishes between the contrast in the intravascular and the interstitial space presumably related to renal excretion and distribution into other spaces such as bone and synovial fluid over the 60min observation time [29]. However, the increase of the partition coefficient between 10 and 30 min after contrast administration, which would usually be the time span where T1 mapping would be acquired in a clinical protocol, was only 0.01. Therefore the difference might not be clinically relevant.

A limitation of the study is the fact that we tested only for the effect of Ibuprofen on T1 time. Other substances with high protein binding capacity such as Diltiazem, Propranolol, Itraconazole or Sufentanil were not evaluated [27]. However, we intended to test a substance that is easily available without prescription, not harmful to volunteers and commonly used. Another limitation might be the fact that we studied the effect of a protein binding substance on the partition coefficient but not on the ECV. However, since calculation of the partition coefficient and ECV is identical, expect for the fact that the latter is multiplied by a constant (1-hematocrit), this should be irrelevant regarding the evaluation of the effect of Ibuprofen.

## Conclusions

In conclusion, contrast enhanced T1 mapping of Gd-BOPTA is unaffected by co-medication with the protein binding substance Ibuprofen in vitro and in vivo and has an excellent reproducibility.

## Competing interests

One of the co-authors, JMF, is a consultant for Guerbet Pharmaceutical Company Switzerland.

## Authors’ contribution

NK: study design, data acquisition, data analysis, data interpretation, manuscript drafting; FS: study design, data acquisition, data analysis, data interpretation, manuscript drafting; TH: data acquisition, manuscript revision; JMF: study design, phantom preparation, data interpretation, manuscript revision; JB: principal investigator, study design, data interpretation, manuscript revision. All authors read and approved the final manuscript.
